# Patient-Reported Outcome-Based, Comprehensive Assessment of Quality of Life of Adults with Congenital Heart Disease in India

**DOI:** 10.5334/gh.1479

**Published:** 2025-10-16

**Authors:** Navaneetha Sasikumar, Sakthi Saravanan, Krishna Prasenan, Seeja Raji, Georg Gutjahr, Abish Sudhakar, Shanthi Chidambarathanu, Philip Moons, Raman Krishna Kumar

**Affiliations:** 1 Department of Pediatric Cardiology, Amrita Institute of Medical Sciences, Kochi, Kerala, India; 2 Department of Health Sciences Research, Amrita Institute of Medical Sciences, Kochi, Kerala, India; 3 T & M Caring Hearts Clinic, Chennai Dr. Mehta’s Children’s Hospital, Chennai, India; 4 KU Leuven Department of Public Health and Primary Care, KU Leuven – University of Leuven, Leuven, Belgium; 5 University of Gothenburg Centre for Person-Centred Care (GPCC), Sahlgrenska Academy, University of Gothenburg, Gothenburg, Sweden; 6 Department of Paediatrics and Child Health, University of Cape Town, Cape Town, South Africa

**Keywords:** adult congenital heart disease, quality of life, patient-reported outcomes, low- and middle-income countries

## Abstract

**Background:**

The improved survival of patients with congenital heart disease (CHD) mandates a shift in focus towards an understanding of patient perspectives on outcomes, particularly focused on quality of life (QOL). This study represents the first systematic, prospective, comprehensive, patient-reported outcome measure (PROM) based QOL assessment of adult congenital heart disease (ACHD) patients in India.

**Methods:**

PROM data from the Indian cohort of APPROACH-IS (Assessment of Patterns of Patient-Reported Outcomes in Adults with Congenital Heart disease – International Study) I (2014) and II (2022)—prospective, cross-sectional international studies conducted at two major centers—were collated. PROMs that were recorded included the determinants of QOL, including physical component summary (PCS) and mental component summary (MCS) of the 12-item health survey, EQ VAS (EuroQoL Visual Analogue Scale), as well as the linear analog scale for assessing QOL (LAS-QOL). The influence of demographic and medical factors on PROMs and various aspects of QOL was assessed with multiple linear regression using the Wilson and Cleary model and generalized estimating equations.

**Results:**

The number of patients studied was 325 (26.71 ± 8.66 years, 56.3% males). More than half had college education, 32.4% had a full-time job, and 26.5% had a partner. Defect complexity was simple in 29.9%, moderate in 37.5%, great in 41.5%, and 77.5% had undergone at least one procedure as part of their treatment. Overall, PROMs from India—particularly the physical domain—fared worse than the global data. Nevertheless, there was improvement from 2014 to 2022. Positive predictors of PROMs included self-reported NYHA (New York Heart Association) class, male sex, younger age, education, and center/year of study. Women reported significantly worse PROMs.

**Conclusion:**

ACHD patients from India report overall excellent PROMs including QOL, despite the majority having complex heart defects. Physical functioning is a key deficiency. Age- and gender-sensitive health policies, systematic early implementation of personalized physical activity training programs, and integration of mental health into cardiac follow-up merit strong consideration.

## Introduction

With significant advancements in diagnosis, interventions, surgery, and post-operative care, more than 90% of children with CHD survive to adulthood in the current era, particularly in high-resource settings (1,2). This has led to a distinct population of ACHD patients who are growing simultaneously in age and number (3). All these patients require lifelong follow-up, many requiring medications and repeat interventions. This can potentially impact their physical and psychological well-being and overall quality of life. The situation in India and other similar low- and middle-income countries is unique in that ACHD survivors who received timely care coexist with another group consisting of late-operated and un-operated CHD.

There has been a growing emphasis on patient-reported outcome measures (PROMs) and patient-reported experience measures (PREMs) for advancing provision of care (4,5). The APPROACH-IS evaluated PROMs in ACHD patients from 15 countries. Patients from India reported one of the lowest scores, not only in physical functioning, but also in the composite score, including psychological health and overall QOL (6,7,8). The APPROACH-IS II was a follow-up study with 8415 patients across 32 countries, which expanded PROMs and added PREMs to the assessment (9,10). Indian data consistently demonstrated poor performance compared to global averages (6,10). No dedicated analysis has been done so far to assess the factors determining these poor outcomes of ACHD patients in India. This study aimed to fill this gap by analyzing the combined Indian data from APPROACH-IS I and II. The results of such an analysis can inform clinical decision-making and guide health policy.

The study questions were: (a) How do ACHD patients in India report their own physical and psychological functioning, health status, and QOL? (b) How are these influenced by demographic, clinical, and healthcare system factors?

## Methods

Study design: Both APPROACH-IS I and II were prospective, cross-sectional descriptive studies. A detailed description of the rationale and methodology of APPROACH-IS I and II has been published previously (7,9). The work presented here investigates the combined Indian data from APPROACH-IS I and II (6,7,8,9,10).

Variables and measurement: Respecting the many determinants of QOL, a combination of tools was resorted to for a comprehensive assessment. The patients’ own perceptions of QOL as such were assessed using the LAS-QOL in APPROACH-IS I and II (11). The patients’ self-report of their health status, another determinant of QOL, was done using EQ VAS (9). The LAS is a vertical tool designed to measure an individual’s overall quality of life (LAS-QOL) and health status (EQ VAS). Patients mark their own assessment of QOL and health status (HS) on a vertical line. It spans from 0 to 100, with 0 signifying the worst imaginable QOL/HS and 100 denoting the best imaginable QOL/HS. The validity, reliability, and responsiveness of the LAS in adults with CHD have been evaluated previously (11).

To gain an in-depth understanding of physical and psychological functioning, which also influence QOL, the 12-item health survey was utilized (12). The SF-12 (Short Form-12) is a shorter version of SF-36/RAND 36. There are 12 questions in SF-12 that cover eight domains of health, including physical functioning, role limitations due to physical health, bodily pain, general health perceptions, vitality, social functioning, role limitations due to emotional problems, and mental health. Two scores were generated based on algorithms using specialized software: the PCS and the MCS. The scores were on a continuous scale from 0 to 100. Higher scores indicate higher physical functioning and mental health. All questionnaires were adapted to the local language.

Study setting: APPROACH-IS I (2013–2015) and II (2021–2022) were carried out in two of the leading pediatric cardiac centers in South India, APPROACH-IS I in center 1 and APPROACH-IS II in center 2. Both centers have been offering pediatric cardiac surgeries and interventions for over two decades. Many of the pediatric patients treated at these centers have now transitioned into adulthood after receiving care as children. These individuals have been meticulously followed up at these centers, forming the foundation for the study.

Procedures: Necessary institutional ethics board permissions were obtained from the primary coordinating site as well as the participating sites. The study was introduced to all adult patients with CHD (operated or unoperated) who presented to the outpatient department by the investigating physician. After informed consent, the patient questionnaire was introduced to the participants in their preferred language. A trained research assistant assisted the patients in answering the questions whenever required. The investigating physician was available for clarification of any points or to address any concerns as the patient filled up the questionnaire. Complete confidentiality of all patient-related identifiers was maintained.

Data was entered into the online data management system REDCap (Research Electronic Data Capture) by a trained research assistant (13). This was created and maintained by the central coordinating center at the University of Leuven in Leuven, Belgium. A unique study ID was provided for each patient and only de-identified data was passed on to the core committee. From the central database of APRROACH IS I and II, data from India was accessed for the study.

Inclusion and exclusion criteria: For inclusion in the study, CHD was defined as ‘a gross structural abnormality of the heart and/or intra-thoracic great vessel that is actually or potentially of functional significance, including defects of mild, moderate, and great complexity’ (14). Other inclusion criteria were being 18 years of age or older at the moment of study inclusion, diagnosed with CHD before the age of 10 years, follow-up at an ACHD center or included in a national/regional registry, physical, cognitive, and language abilities to complete self-report questionnaires.

Variables: Demographic data included age, sex, marital status, number of children, education level, and employment status based on self-report. Medical data collected from charts included diagnosis of CHD, history of cardiac surgeries or interventions, number of cardiac admissions (over the past five years), number of cardiac outpatient visits (over the past five years), and NYHA functional classification system (self-reported). PROMS, as mentioned above, were also collected.

### Statistical methods

For demographic characteristics, continuous variables were summarized by median and IQR (Interquartile range). Categorical variables were summarized by frequencies and percentages. PROMs were standardized by calculating Z scores for data sets from APPROACH-IS I and II.

Relationships between the PROMs were visualized using pairs plots. Dependencies of PROMs on demographic characteristics and medical variables were explored visually by box plots. For comparing variables between men and women, the Wilcoxon rank-sum test and Pearson’s Chi-squared test were applied. Differences in PROMs between men and women were visualized by side-by-side box plots, stratified by employment status. Pearson’s test was used to ascertain the correlation between disease complexity rank and PROMs. An independent sample t-test was used to compare the PROMs by dichotomous variables. A one-way ANOVA with a post hoc Bonferroni test was used to compare the PROMs among self-reported NYHA class, complexity of disease, education, and occupation.

**Figure 1:**
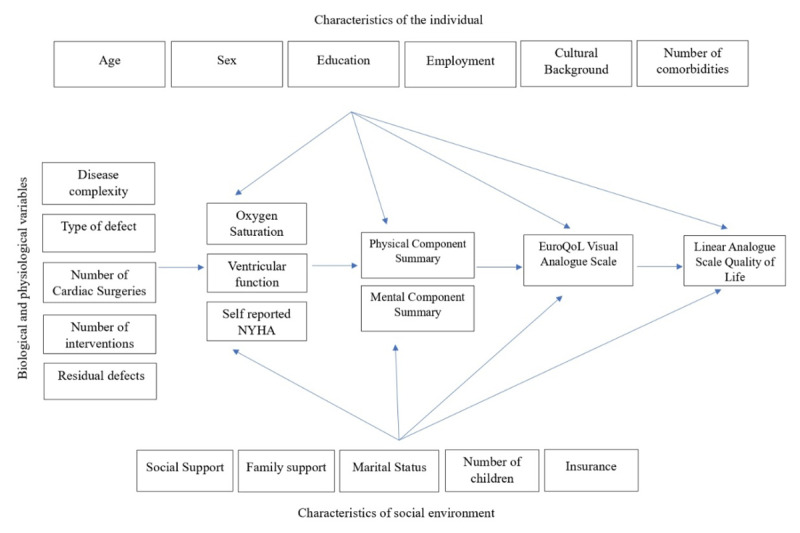
The conceptual model of Wilson and Cleary, eluding causal links among characteristics of the individual, social environment, biological and physiological variables contributing to various facets of quality of life assessment. Characteristics of the individual, the underlying disease, and the environment affect physiological factors. The physiological factors influence the physical and psychological functioning, which in turn influences the patient’s own perception of their health status and overall quality of life.

The Wilson and Cleary model was resorted to to delineate the predictors of PROMs including QOL in a meaningful way (15,16). In order to respect the complex construct of QOL, a comprehensive framework was used to promote the selection of appropriate variables and to identify potential links between the variables within this complex construct. This model, developed by Wilson and Cleary, is a taxonomy of patient outcomes that links the characteristics of the individual to that of the environment, all of which can influence QOL (Figure 1) (17,18). When there are multiple determinants (PCS, MCS, LAS-HS) of an outcome (LAS-QOL), and the determinants themselves are influenced by various predictors, the model provides a comprehensive framework to analyze and report. Four independent forward stepwise multiple linear regressions were performed to predict the different PROMs (17,18). Variables that could influence each of the PROMs were ranked by partial R^2^ order. The T-values of each predictor show whether the corresponding regression coefficient is significantly different from zero, meaning we test the null hypothesis that the coefficient equals zero. An absolute T value of 2 or greater is considered statistically significant (p-value less than 0.05). This would suggest that the predictor’s coefficient is two standard errors away from zero.

To elucidate the magnitude of contribution of each variable to the explained variance, Cohen’s f^2^ was calculated (19,20). Cohen’s f^2^ is a measure of effect size for multiple regression models, particularly when assessing the incremental effect of adding a new predictor. Cohen suggests f^2^ values of 0.02, 0.15, and 0.35 for small, medium, and large effect sizes, respectively (17). The average rank and effect size of each variable across the different PROMs were calculated to provide an all-encompassing assessment of a complex scenario (17,18).

As multiple linear regression estimates the effect of each predictor on the outcome, assuming that all other variables are held constant and that there is no within-group correlation, Generalized Estimating Equations (GEE) analysis was also done. The latter models correlation structure within clusters, and provides more accurate standard errors and significance tests for correlated data.

Statistical analyses were conducted using SPSS Version 20.0 for Windows (IBM Corporation ARMONK, NY, USA) and R Version 4.4.0.

## Results

Baseline characteristics: A total of 325 patients participated in APPROACH-IS I (n = 200) and II (n = 125) from India. Relevant demographic and medical details are organized in Table 1, stratified by gender. The majority of patients [252 (77.5%)] had undergone at least one procedure as part of treatment for their CHD. A large number of patients [73 (22.5%)] had neither undergone an interventional nor a surgical procedure.

**Table 1:** Demographics and medical details stratified by gender.


CHARACTERISTIC	OVERALL, N = 325^1^	MEN, N = 183^1^	WOMEN, N = 142^1^	p-value^2^

Age	24 (21, 30)	24 (21, 31)	24 (21, 29)	0.7

Disease Complexity				0.2

Simple	68 (21%)	32 (17%)	36 (25%)	

Moderate	122 (38%)	74 (40%)	48 (34%)	

Complex	135 (42%)	77 (42%)	58 (41%)	

Employed	182 (56%)	119 (65%)	63 (44%)	<0.001

Education				0.7

Below high school	27 (8.3%)	16 (8.7%)	11 (7.7%)	

High school	95 (29%)	49 (27%)	46 (32%)	

Bachelor’s degrees	121 (37%)	71 (39%)	50 (35%)	

Master’s degree	82 (25%)	47 (26%)	35 (25%)	

Married	86 (26%)	44 (24%)	42 (30%)	0.3

No of children				0.004

0	268 (82%)	150 (82%)	118 (83%)	

1	23 (7.1%)	7 (3.8%)	16 (11%)	

2	28 (8.6%)	22 (12%)	6 (4.2%)	

3	4 (1.2%)	2 (1.1%)	2 (1.4%)	

4	2 (0.6%)	2 (1.1%)	0 (0%)	

Self-reported NYHA				0.8

1	161 (50%)	94 (52%)	67 (48%)	

2	110 (34%)	62 (34%)	48 (34%)	

3	33 (10%)	17 (9.4%)	16 (11%)	

4	17 (5.3%)	8 (4.4%)	9 (6.4%)	

Unknown	4	2	2	


^1^ Median (IQR); n (%). ^2^ Wilcoxon rank sum test; Pearson’s Chi-squared test; Fisher’s exact test.

PROMs: Table 2 shows the characteristics of the PROM assessments. The global data of APPROACH-IS I and II showed an overall worsening of PROMS from 2014 to 2022. On comparison with global data, India fared better in the data from 2022 (Table 2). Table 2 also shows how the two centers fared with respect to the PROMs assessed. PCS, MCS, and EQ VAS were comparable between the two centers. There was a difference in LAS-QOL between the two centers, which was found to be statistically significant. Center 1 reported better QOL on the LAS-QOL scale compared to center 2 (76.67 ± 18.52 versus 72.10 ± 18.26, p = 0.031).

**Table 2:** Comparison of Patient Reported outcome measures with global data.


CHARACTERISTIC^1^ MEAN (SD)	2014, N = 200^1^	GLOBAL DATA 2014, N = 4028	p VALUE	2022, N = 125^1^	GLOBAL DATA 2022, N = 8415	p VALUE

PCS	67.2 (20.7)	77.2 ± 20.9	<0.001	68.8 (20.5)	75.2 ± 21.3	0.001

MCS	68.9 (19.5)	72.1 ± 19.0	0.021	71.4 (17.7)	69.2 ± 18.7	0.166

EQ VAS	77.6 (18.7)	77.9 ± 16.5	0.831	76.3 (16.1)	73.3 ± 17.8	0.038

QOL	76.7 (18.5)	78.3 ± 16.6	0.216	72.1 (18.3)	72.5 ± 20.0	0.809


*PCS – Physical Component Summary*. *MC-S – Mental Component Summary*. *EQ VAS – EuroQoL Visual Analogue Scale*. *QoL – Quality of Life*.

**Figure 2:**
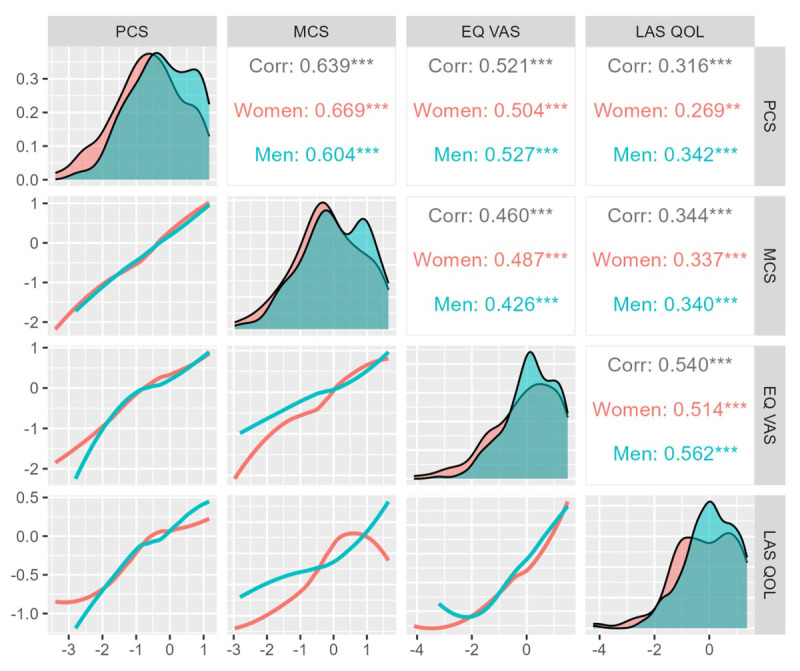
Correlation of the various patient-reported outcome measures. All measures were strongly related to each other. This relationship remained similar for men and women. Standardized values of each of these PROMs are depicted on the x and y axes to demonstrate the relationship between them.

**Figure 3:**
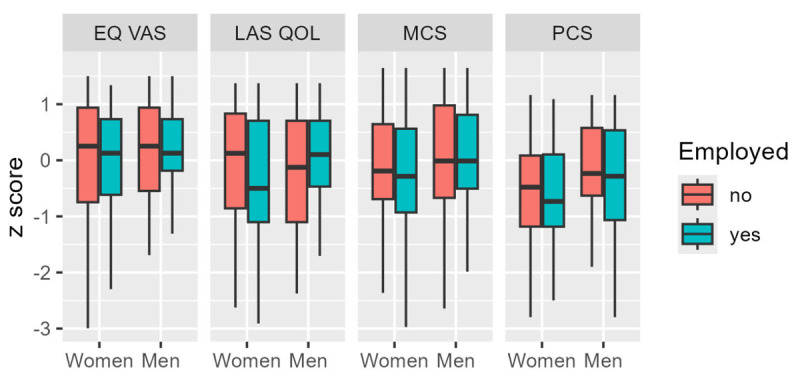
Differential effect of unemployment on the various patient-reported outcome measures. Employed women had worse scores in EuroQoL VAS, MCS, and LAS-QOL. PCS scores of women were unaffected by employment status.

PROMs were significantly worse for women than for men in both the physical and psychological domains. Men and women were of comparable age, disease complexity, and self-reported NYHA class but had significantly different QOL [PCS (71.46 ± 19.12 versus 63.11 ± 21.56, p < 0.001); MCS (71.70 ± 18.36 versus 67.47 ± 19.24, p = 0.044); LAS-QOL (76.81 ± 16.68 versus 72.44 ± 20.47, p = 0.040); and EQ VAS (78.96 ± 15.50 versus 74.69 ± 20.10, p = 0.0380; Supplemental Tables 1–4]. Figure 2 shows that all QOL measures were related to each other to a modest degree and that this relation was similar for men and women. Employed women reported worse physical and mental health and self-reported QOL (Figure 3). Such a differential effect was not seen in men. Other demographic variables like age and education did not show a similar effect modification.

Univariate analysis: Consistently throughout the four PROMs assessed—PCS, MCS, LAS-QOL, and EQ VAS—there was a variation in mean values within the self-reported NYHA classes, p < 0.001 (Supplemental Table 1). Better self-reported NYHA class was associated with better PROM scores. PCS was associated with a higher number of variables compared to the other PROMs on univariate assessment (Supplemental Table 1). Patients with simple heart defects had a higher PCS compared to those with heart diseases of moderate and great complexity (72.26 ± 20.01 versus 68.92 ± 19.96 versus 64.58 ± 21.11, p = 0.032). Younger age correlated with a better PCS (Pearson correlation coefficient –0.165, p = 0.003). Patients who had undergone catheter-based treatment had a higher PCS compared to those who had had no procedure, as well as those who had undergone open heart surgeries (70.25 ± 18.48 versus 63.02 ± 21.52 versus 69.96 ± 20.50 p = 0.046). Neither age, nor complexity of heart defect, nor previous procedures showed correlation with any of the other PROMs measured (Supplemental Tables 2, 3, 4). None of the PROMs showed any significant difference with respect to job status and marital status. Higher educational status showed an association with better PCS (p < 0.001) (Supplemental Table 1) and a higher score on EQ VAS (Supplemental Table 4).

**Figure 4:**
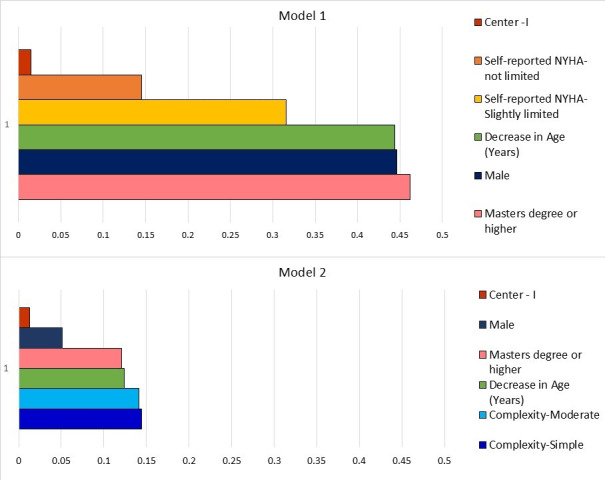
Hierarchy of factors influencing overall quality of life in order of effect size from the Wilson and Cleary model. The average effect size is shown on the x-axis and the factors responsible on the y-axis.

Multiple linear regression models: Relative ranking and effect sizes of items measuring physical functioning (PCS), psychological functioning (MCS), self-reported health status (EQ VAS), and overall QOL (LAS-QOL) using adjusted R^2^-independent forward stepwise multiple linear regression are shown in Table 3. The models were weak, as evidenced by the total R^2^ values. Age, sex, and education had large effect sizes based on Cohen’s f^2^ in model 1 (Figure 4). Self-reported NYHA class 1 and 2 were predictive of physical and psychological functioning, influencing self-reported health status and overall QOL, with modest effect size. Centre 1 had a small effect size (Figure 4). Male sex, lower age, and higher education primarily influenced physical functioning. When NYHA was excluded, disease complexity gained significance as a predictor for physical functioning (Table 3, Model II). Average R^2^ ranks could not be considered for comparing the relative importance of various facets of QOL assessment, as some of the variables failed to have significant associations with selected facets of QOL assessment.

**Table 3:** Relative ranking and effect sizes of items measuring physical functioning, psychological functioning and general health status and Quality of Life using adjusted R^2^-independent forward stepwise multiple linear regression.


MODEL 1 : NYHA INCLUDED											

VARIABLES	PCS (TOTAL R^2^ = 0.339)		MCS (TOTAL R^2^ = 0.123)		HS (TOTAL R^2^ = 0.134)		QOL (TOTAL R^2^ = 0.040)		AVERAGE R^2^ RANKS	t VALUE	AVERAGE EFFECT SIZE (COHEN’S f^2^)
			
	PARTIAL R^2^ RANK	EFFECT SIZE (t-VALUE)	PARTIAL R^2^ RANK	EFFECT SIZE (t-VALUE)	PARTIAL R^2^ RANK	EFFECT SIZE (t-VALUE)	PARTIAL R^2^ RANK	EFFECT SIZE (t-VALUE)			

Self-reported NYHA-not limited	5 (0.090)	9.3	1 (0.035)	6.7	2 (0.057)	6.1	2 (0.026)	2.9	2.5	6.25	0.145

Self-reported NYHA-Slightly limited	1 (0.009)	2.9	–	–	1 (0.019)	2.5	–	–	1	2.7	0.316

Male	3 (0.044)	3.8	–	–	–	–	–	–	3	3.8	0.446

Masters degree or higher	2 (0.034)	3.3	–	–	–	–	–	–	2	3.3	0.462

Center -I	–	–	–	–	–	–	1 (0.006)	2.5	1	2.5	0.015

Decrease in Age (Years)	4 (0.045)	3.4	–	–	–	–	–	–	4	3.4	0.444

**MODEL 2: NYHA EXCLUDED**											

	**(TOTAL R^2^ = 0.136)**		**(TOTAL R^2^ = 0.012)**		**(TOTAL R^2^ = 0.014)**		**(TOTAL R^2^ = 0.014)**				

Male	3(0.027)	4.3	1(0)	2.0	1(0)	2.2	–	–	1.7	2.83	0.051

Decrease in Age (Years)	4(0.029)	4.2	–	–	–	–	–	–	4	4.2	0.124

Masters degree or higher	5(0.031)	3.2	–	–	–	–	–	–	5	3.2	0.121

Complexity-Simple	1(0.011)	3.7	–	–	–	–	–	–	1	3.7	0.144

Complexity-Moderate	2(0.015)	2.2	–	–	–	–	–	–	2	2.2	0.141

Center – I	–	–	–	–	–	–	1(0.0001)	2.2	1	2.2	0.013


PCS – Physical Component Summary. MCS – Mental Component Summary. EQ VAS – EuroQoL Visual Analogue Scale. QoL – Quality of Life.

GEE Model: Self-reported NYHA class 1 (not limited) had the highest influence on PCS [regression coefficient 23.34 (14.67–32.02), p < 0.001]; MCS [15.72 (7.25–24.18), p < 0.001]; and LAS-HS [17.27 (7.61–26.93), p < 0.001]. Higher education [15.37 (7.88–22.86), p < 0.001]; male sex [7.90 (4.18–11.62), p < 0.001]; and decrease in age [0.37 (0.16–0.58), p = 0.001] were associated with better physical functioning, similar to regression models. In addition, simple defects were associated with better PCS scores [5.38(0.28–10.47), p < 0.039]. Self-reported NYHA class 1 [17.27 (7.61–26.93), p < 0.001]; higher education [13.89 (7.24–20.55), p < 0.001]; and male sex [3.71 (0.02–7.40), p = 0.049] were also predictive of better scores on LAS-HS. Center 1 retained its predictive effect on LAS-QOL in the GEE model [4.74 (0.62–8.85), p < 0.024] (Supplemental Table 5).

## Discussion

This study represents the first comprehensive, prospective PROM data among ACHD patients in India. PROM data from this study indicates that the current cohort of ACHD patients in India enjoys excellent overall patient-reported outcomes. The use of multiple scoring systems enhances the study’s credibility. Self-reported health status among ACHD patients in this study exceeds that of the average Indian population, as reported in a recent study (21). Considering that nearly 80% of this cohort had CHD of moderate-severe complexity, this is remarkable. Interestingly, the lowest values observed among the various PROMs assessed were in the physical domain of the SF-12 survey. Despite rating their health status highly with an EQ VAS score of 77.11 ± 17.74 (where 100 indicates perfect health), this cohort of ACHD patients in India reported poorer physical functioning compared to their mental functioning and LAS-QOL scores. This finding aligns with previous studies (22,23) and is consistent with global data from the APPROACH-IS I, where India had the lowest PCS scores among all participating countries, significantly below the global average. India also reported the lowest composite APPROACH-IS I score, which integrates various PROMs including the PCS and MCS, EQ VAS, and LAS-QOL, among others. This trend persisted from 2014 to 2022. However, in the more recent APPROACH-IS II data, India showed relatively better performance in PROMs (Table 2) (10). APPROACH-IS II data showed that despite patients from high-income countries being older, they reported better physical and mental health and QOL compared to low- and middle-income countries. Gross national income per capita cannot be easily altered in the short term. Hence, identifying modifiable factors responsible for QOL can help develop relevant interventions within the resource constraints prevalent in low- and middle-income countries.

Younger age and less complex heart defects correlated with higher scores in the physical domain according to univariate analysis. The GEE model also showed that simple defects are modestly predictive of better physical functioning. Similarly, patients who had undergone catheter interventions demonstrated better physical domain scores compared to those who had undergone no procedures or surgeries, on univariate analysis, aligning with previous findings (23) highlighting the negative impact of surgeries. Interestingly, these factors did not significantly influence other PROMs, including the mental domain of SF-12, EQ VAS, and LAS-QOL. Employment and marital status also showed no significant influence on any PROMs. Age retained significance in the regression and GEE models for PCS, contrary to the non-significant effect observed for the type of intervention. Previous studies have reported conflicting findings regarding the influence of age (22,23).

Intuitively, despite overall low PCS values compared to global data, those ACHD patients with self-reported NYHA class 1 and 2 performed well in both the physical (PCS) and mental (MCS) domains of the SF-12, self-reported health status (EQ VAS), as well as LAS-QOL, with decreasing effect size in sequence in the Wilson and Cleary linear regression models. What is significant is that this is irrespective of medical factors including complexity of defect and number of surgeries or interventions undergone. Model I and II corroborate this aspect, whereby self-reported NYHA negates the effect of disease complexity on QOL. Self-reported NYHA class (not limited) was the strongest and most consistent predictor of PROMs in the GEE model as well. Even more importantly, functional capacity is a modifiable factor; it presents a unique opportunity to implement personalized physical activity training programs for these patients, aiming to further enhance their overall QOL. Previous reports on the influence of functional capacity on QOL have been inconsistent, highlighting the need for further research in this area (22,23). The correlations observed among QOL measures suggest that improving physical capacity could potentially bolster both psychological functioning and overall QOL.

Another factor that influenced QOL was the non-modifiable factor of gender. Despite being of comparable age, disease complexity, and self-reported NYHA class, men consistently exhibited better PROMs including QOL. This trend was most pronounced and statistically significant in the physical domain, as confirmed by the linear regression and GEE models. This gender difference has been consistently observed in the literature (22,23). The reasons for this can only be speculated. An interaction between biological and psychosocial factors can possibly explain this gender disparity, similar to findings in other clinical settings (24). Sex hormones affecting cardiovascular physiology and altering mood and pain perception, as well as risks related to reproductive health—including pregnancy and contraception—could negatively influence QOL in women. Differences in coping styles and greater gender role strain as discussed below could also potentially contribute to this (25). Further exploration revealed that employment had a negative impact on the PROMs for women, while the opposite effect was observed for men. Notably, this effect modification was limited to employment and did not extend to other factors. Traditional Indian culture places the major burden of domestic and caregiving responsibilities on women, irrespective of employment status. On the other hand, societal support in the form of institutional care for children and the elderly is less prevalent. Performance pressure at both fronts, without adequate support, can be presumed to negatively influence the QOL of employed women (26).

Despite comparable self-reported health status and similar scores in the physical and mental domains of the 12-item health survey, there was a statistically significant difference in self-reported QOL between the centers/time periods. This difference remained significant in the linear regression and GEE models. Although the simplicity of the LAS-QOL scale and its alleged responsiveness to subjective mood are considered as limitations, these can perhaps be considered its core strengths as well. QOL is a dynamic and perception-driven construct. The scale’s responsiveness to change in the socio-cultural macro-environment makes it relevant in time-sensitive, real-world evaluations. As shown in this study, interpretation may vary across cultures and time periods. This could potentially reflect the lived experience of the patients rather than measurement error. Furthermore, data from center 2 was collected in the post-COVID era, a time when the prevalence of anxiety and depression had increased in the general population, which may have influenced the findings (27,28). The fact that global data itself showed lower scores in 2022 compared to 2014 supports this possibility (Table 2).

The Wilson and Cleary model is considered robust as a conceptual framework to characterize predictors of health-related QOL (29). Model I was stronger, suggesting that among the variables studied, self-reported NYHA is a strong predictor explaining a significant part of the variance in QOL. Once NHYA is taken out of the modeling, disease complexity shows a statistical significance. Thus, self-reported NYHA masked or suppressed the effect of disease complexity. However, the R^2^ falls because disease complexity can’t explain as much of the variance in QOL as self-reported NYHA did. The effect of disease complexity is perhaps mediated by self-reported NYHA. In other words, patients with underlying diseases of great complexity can report higher QOL scores when their self-reported functional class is better. The global APPROACH-IS II data also concluded that PROMs may not correlate with disease complexity (30). The Wilson and Cleary model has been able to explain between 22.9% to 72% of the variance in overall QOL in various other clinical contexts (29). High R^2^ values in the range of 0.66 to 0.69 have been reported in previous QOL studies (18).

The study provides valuable information which can improve clinical care. Encouraging better education and emphasizing the importance of physical training right from childhood can positively influence outcomes. The elderly and women will need more attention and personalized care. From a public health perspective, the findings have significant implications. Long-term, multi-specialty integrated models of care that look beyond procedural success and survival are needed for enabling ACHD patients to have optimal QOL. Gender disparities despite comparable clinical profiles speak volumes on the sociocultural and occupational disadvantages that disproportionately affect women in low- and middle-income countries. Age had a similar effect as well. Gender- and age-sensitive health policies are a necessity to address the broader issues that lead to such disparities. The fact that temporal and inter-center differences could possibly have been influenced by post-pandemic mental health issues reinforces the role of integrating mental health into routine cardiac follow-up. Overall, the findings offer a groundwork for formulating evidence-based health policy and designing contextual PROM-driven interventions for ACHD patients in India.

## Limitations

The cross-sectional design of the study limits causal inferences. The data set from two centers were collected during different time periods as part of two large international multicenter studies. The era of data collection could have influenced the results. The two centers cater to patients primarily from two different states in South India, managed by two different teams. Although general principles remain the same, patient care approaches may vary between the centers, which could perhaps influence PROMs. The GEE model was used to account for this clustering. The generalizability of the findings to other parts of India and/or other low- and middle-income country settings could be limited by the sociocultural background that largely prevails in these southern states. Both these states fall into the high human development index category, whereas the majority of India belongs to the medium category (31).

The multiple linear regression analysis models were weak as such, suggesting the role of factors beyond those assessed in the study, on QOL. The low R^2^ values corroborate this possibility.

Self-reported NYHA reflects the patients’ perspective and is more subjective compared to physician-rated NYHA class. It could be influenced by the patient’s own perception of physical activity, memory, and mood. Self-reported NYHA needs to be interpreted in the light of the fact that many of the ACHD patients may potentially have adjusted their physical activities over the years to what is achievable for them.

## Conclusion

This study represents the first systematic, prospective PROM-based assessment of QOL of ACHD patients in India, utilizing a battery of tools. The study demonstrates excellent overall PROMs, notwithstanding the fact that nearly 80% had defects of moderate or great complexity. The primary factors influencing QOL were self-reported NYHA class, gender, age, education, and the center of assessment. The physical domain emerged as the most compromised aspect of PROMs. These findings highlight a unique opportunity to implement personalized physical activity training programs for these patients, aiming to further enhance their overall QOL. This would have to start early to negate the effect of age and would be particularly important for women who have a disproportionately low QOL. There is a strong case for integrating mental health into routine cardiac follow-up as well.

## Data Accessibility Statement

Data will be maintained for five years after publication.

## Additional File

The additional file for this article can be found as follows:

10.5334/gh.1479.s1 Supplementary File. Supplemental Tables 1 to 5.
