# Supplementary material for: Patient-Reported Outcome-Based, Comprehensive Assessment of Quality of Life of Adults with Congenital Heart Disease in India
Source: Glob Heart. 2025 Oct 16;20(1):96. doi: 10.5334/gh.1479 (PMC12533435; doi:10.5334/gh.1479)
Supplement: Supplementary File. — Supplemental Tables 1 to 5. [file gh-20-1-1479-s1.pdf]

Supplemental Table -1 Association of variables with Physical Component Summary

Variables	category	n	Mean ± SD	P value	Subgroup difference
Gender	Male<br>Female	183<br>142	71.46 ± 19.12<br>63.11 ± 21.56	<0.001	NA
Age	Correlation coefficient (r)	323	-0.165	0.003	
Center	Chennai<br>Kochi	200<br>125	67.21 ± 20.73<br>68.78 ± 20.47	0.505	
Self report of NYHA	a) Not limited<br>b) Slightly limited<br>c) Considerably limited<br>d) Very Limited	161<br>110<br>33<br>17	78.07 ± 16.59<br>60.48 ± 18.74<br>51.78 ± 16.91<br>48.93 ± 23.39	<0.001	a>b,c,d p<0.001<br>b>c, p=0.085<br>b>d, p=0.079<br>c>d, p=1.000
Complexity of heart defect	a) Simple<br>b) Moderate<br>c) Complex	68<br>122<br>135	72.26 ± 20.01<br>68.92 ± 19.96<br>64.58 ± 21.11	0.032	a>b, p=0.845<br>a>c, p=0.036<br>b>c, p=0.271
Complexity rank	Correlation coefficient (r)		0.157	0.005	NA
Do you have children	No<br>Yes	268<br>57	67.76 ± 20.90<br>68.11 ± 19.58	0.909	NA
Number of children	No<br>1<br>2	268<br>23<br>28	67.75 ± 20.86<br>71.05 ± 14.60<br>69.80 ± 22.33	0.693	NA
Education	a) Did not complete high school<br>b) High school / diploma/ secondary education<br>c) Bachelors degree<br>d) Masters degree or higher	27<br>95<br>121<br>82	54.20 ± 20.90<br>65.57 ± 21.69<br>69.10 ± 18.54<br>73.00 ± 20.18	<0.001	a<b, p=0.060<br>a<c, p=0.003<br>a<d, p<0.001<br>b<c, p=1.000<br>b<d, p=0.089<br>c<d, p=1.000
Marital status	Never married<br>Married or living with partner	238<br>86	68.82 ± 20.70<br>65.13 ± 20.36	0.156	NA
Currently student	a) No<br>b) Yes part time<br>c) Yes full time	186<br>30<br>105	65.76 ± 20.99<br>62.00 ± 20.49<br>72.83 ± 19.28	0.005	a>b, p=1.000<br>a<c, p=0.014<br>b<c, p=0.032
Current work situation	Full-time paid job<br>Part-time paid job<br>Homemaker<br>Job seeking<br>Unemployed<br>Other	105<br>12<br>23<br>32<br>111<br>39	70.33 ± 19.73<br>63.33 ± 17.75<br>61.00 ± 22.98<br>72.68 ± 18.42<br>66.29 ± 21.30<br>67.72 ± 21.72	0.221	NA

Number of interventional catheterizations/ Number of cardiac surgery	No procedure<br>Cath<br>Surgery	73<br>25<br>185	63.02 ± 21.52<br>70.25 ± 18.48<br>69.96 ± 20.50	0.046	NA

Supplemental table 2 Association of variables with Mental Component Score					
**Variables**	**category**	**n**	**Mean ± SD**	**P value**	**Subgroup difference**
Gender	Male<br>Female	183<br>142	71.70 ±18.36<br>67.47±19.24	0.044	NA
Age	Correlation coefficient (r)	323	-0.022	0.697	
Center	Chennai<br>Kochi	200<br>125	68.89 ± 19.51<br>71.40 ± 17.66	0.243	
Self report of NYHA	a) Not limited<br>b) Slightly limited<br>c) Considerably limited<br>d) Very Limited	161<br>110<br>33<br>17	76.51±17.31<br>65.13±17.32<br>59.12±18.96<br>57.90±20.10	<0.001	a>b,c,d p<0.001<br>b>c, p=0.522<br>b>d, p= 0.701<br>c>d, p= 1.000
Complexity of heart defect	a) Simple<br>b) Moderate<br>c) Complex	68<br>122<br>135	70.33±16.42<br>70.97±19.79<br>68.61±19.14	0.590	NA
Complexity rank	Correlation coefficient (r)		0.081	0.148	NA
Do you have children	No<br>Yes	267<br>57	69.39±19.16<br>72.00±17.43	0.343	NA
Number of children	No<br>1<br>2	268<br>23<br>28	69.39±19.12<br>72.55±17.47<br>73.36±17.83	0.456	NA
Education	a) Did not complete high school<br>b) High school / diploma/ secondary education<br>c) Bachelors degree<br>d) Masters degree or higher	27<br>95<br>121<br>82	65.20±18.79<br>68.95±19.45<br>70.60±18.46<br>71.34±18.72	0.465	NA
Marital status	Never married<br>Married or living with partner	238<br>86	69.68±19.10<br>70.58±18.14	0.704	NA

Currently student	a) No<br>b) Yes part time<br>c) Yes full time	186<br>30<br>105	68.13±18.84<br>67.22±15.22<br>73.32±19.37	0.057	NA
Current work situation	Full-time paid job<br>Part-time paid job<br>Homemaker<br>Job seeking<br>Unemployed<br>Other	105<br>12<br>23<br>32<br>111<br>39	70.96±17.52<br>62.24±18.19<br>67.66±21.54<br>71.78±17.89<br>69.06±19.68<br>70.29±19.68	0.667	NA
Number of interventional catheterizations/ Number of cardiac surgery	No procedure<br>Cath<br>Surgery	73<br>25<br>185	68.26 ± 18.69<br>70.37 ± 17.47<br>69.68 ± 19.48	0.835	NA

Supplemental Table 3 Association of variables with Linear analog scale - Quality of Life

Variables	category	n	Mean ± SD	P value	Subgroup difference
Gender	Male<br>Female	182<br>141	76.81 ± 16.68<br>72.44 ± 20.47	0.040	NA
Age	Correlation coefficient (r)	321	-0.066	0.236	
Center	Chennai<br>Kochi	200<br>125	76.67 ± 18.52<br>72.10 ± 18.26	0.031	
Self report of NYHA	a) Not limited<br>b) Slightly limited<br>c) Considerably limited<br>d) Very Limited	161<br>109<br>32<br>17	77.64 ± 18.23<br>72.55 ± 18.46<br>71.25 ± 19.05<br>67.94 ± 18.29	0.031	a>b, p=0.158<br>a>c, p=0.442,<br>a>d, p=0.237<br>b>c,d, p=1.000<br>c>d, p=1.000
Complexity of heart defect	a) Simple<br>b) Moderate<br>c) Complex	68<br>121<br>134	74.13 ± 20.53<br>76.29 ± 17.73<br>74.04 ± 18.23	0.582	NA
Complexity rank	Correlation coefficient (r)		0.043	0.440	NA
Do you have children	No<br>Yes	266<br>56	74.55 ± 18.59<br>76.30 ± 18.36	0.521	NA
Number of children	No<br>1<br>2	267<br>23<br>27	74.61 ± 18.58<br>80.65 ± 15.69<br>75.11 ± 20.51	0.327	NA

Education	a) Did not complete high school<br>b) High school / diploma/ secondary education<br>c) Bachelors degree<br>d) Masters degree or higher	27<br>95<br>121<br>80	68.52 ± 21.02<br>74.03 ± 17.34<br>77.21 ± 17.10<br>74.60 ± 20.70	0.150	NA
Marital status	a)Never married<br>b)Married or living with partner	237<br>85	75.51 ± 18.59<br>73.39 ± 18.41	0.367	NA
Currently student	a) No<br>b) Yes, part time<br>c) Yes, full time	185<br>30<br>104	74.11 ± 18.98<br>72.00 ± 16.11<br>76.84 ± 18.43	0.334	NA
Current work situation	Full-time paid job<br>Part-time paid job<br>Homemaker<br>Job seeking<br>Unemployed<br>Other	104<br>12<br>23<br>32<br>111<br>38	75.43 ± 18.51<br>74.58 ± 14.69<br>73.91 ± 19.71<br>72.03 ± 18.66<br>73.45 ± 19.54<br>80.92 ± 15.72	0.350	NA
Number of interventional catheterizations/ Number of cardiac surgery	No procedure<br>Cath<br>Surgery	72<br>25<br>184	73.75 ± 18.51<br>69.72 ± 21.02<br>75.98 ± 18.85	0.258	NA

**Supplemental Table -4 Association of variables with EuroQoL Visual Analogue Scale**					
**Variables**	**category**	**n**	**Mean ± SD**	**P value**	**Subgroup difference**
Gender	Male<br>Female	183<br>140	78.96 ± 15.50<br>74.69 ± 20.10	0.038	NA
Age	Correlation coefficient (r)	321	-0.108	0.054	
Center	Chennai<br>Kochi	198<br>125	77.62 ± 18.73<br>76.31 ± 16.08	0.520	NA
Self report of NYHA	a) Not limited<br>b) Slightly limited<br>c) Considerably limited<br>d) Very Limited	161<br>110<br>32<br>16	82.87 ± 14.29<br>73.68 ± 17.92<br>67.34 ± 19.84<br>60.94 ± 20.91	<0.001	a>b,c,d p<0.001<br>b>c, p=0.347<br>b>d, p=0.026<br>c>d, p=1.000
Complexity of heart defect	a) Simple<br>b) Moderate<br>c) Complex	67<br>122<br>134	78.01 ± 16.51<br>78.60 ± 15.51<br>75.30 ± 20.05	0.299	NA

Complexity rank	Correlation coefficient (r)		0.090	0.109	NA
Do you have children	No<br>Yes	266<br>56	76.92 ± 18.57<br>77.68 ± 13.21	0.720	NA
Number of children	No<br>1<br>2	267<br>22<br>28	76.10 ± 18.57<br>81.82 ± 12.49<br>77.50 ± 12.43	0.473	b>a, p=0.665<br>b>c, p=1.000<br>c>a, p=1.000
Education	a) Did not complete high school<br>b) High school / diploma/ secondary education<br>c) Bachelors degree<br>d) Masters degree or higher	27<br>95<br>121<br>80	64.63 ± 17.43<br>76.07 ± 19.02<br>78.73 ± 15.75<br>80.11 ± 17.54	0.001	a<b, p=0.016<br>a<c, p=0.001<br>a<d, p<0.001<br>b<c, p=1.000<br>b<d, p=0.754<br>c<d, p=1.000
Marital status	Never married<br>Married or living with partner	237<br>85	78.10 ± 17.94<br>74.55 ± 17.01	0.114	NA
Currently student	a) No<br>b) Yes part time<br>c) Yes full time	185<br>30<br>104	75.89 ± 17.29<br>72.06 ± 17.55<br>80.48 ± 18.17	0.029	a>b, p=0.809<br>a<c, p=0.103<br>b<c, p=0.065
Current work situation	Full-time paid job<br>Part-time paid job<br>Homemaker<br>Job seeking<br>Unemployed<br>Other	104<br>12<br>23<br>32<br>111<br>38	77.93 ± 16.63<br>70.83 ± 15.50<br>74.65 ± 18.54<br>77.31 ± 18.82<br>76.28 ± 18.97<br>80.89 ± 16.95	0.536	NA
Number of interventional catheterizations/ Number of cardiac surgery	No procedure<br>Cath<br>Surgery	72<br>24<br>185	73.58 ± 19.19<br>80.37 ± 11.62<br>77.99 ± 17.73	0.128	

**Supplemental Table - 5 Generalized Estimating Equations Model**								
**Parameters**	**Physical Component Summary**		**Mental Component Summary**		**Linear Analog Scale - Health Status**		**Linear Analog Scale - Quality of Life**	
	**Coefficient (95% CI)**	**P value**	**Coefficient (95% CI)**	**P value**	**Coefficient (95% CI)**	**P value**	**Coefficient (95% CI)**	**P value**
Self-reported NYHA-not limited	23.34 (14.67 - 32.02)	<0.001	15.72 (7.25 - 24.18)	<0.001	17.27 (7.61 - 26.93)	<0.001	-	-

High school (or equivalent diploma)/ secondary education	8.41 (1.06 - 15.77)	0.025	-	-	10.79 (4.13 - 17.45)	0.002	-	-
Bachelor’s degree	9.99 (2.77 - 17.21)	0.007	-	-	12.74 (6.43 - 19.05)	<0.001	-	-
Master’s degree or higer	15.37 (7.88 - 22.86)	<0.001	-	-	13.89 (7.24 - 20.55)	<0.001	-	-
Complexity-Simple	5.38 (0.28 - 10.47)	0.039	-	-	-	-	-	-
Male	7.90 (4.18 - 11.62)	<0.001	-	-	3.71 (0.02 - 7.40)	0.049	-	-
Decrease in Age (Years)	0.37 (0.16 - 0.58)	0.001	-	-	-	-	-	-
Center -1	-	-	-	-	-	-	4.735 (0.62 - 8.85)	0.024
